# Uptake and bio-transformation of telmisartan by cress (*Lepidium sativum*) from sewage treatment plant effluents using high-performance liquid chromatography/drift-tube ion-mobility quadrupole time-of-flight mass spectrometry

**DOI:** 10.1007/s11356-021-14289-4

**Published:** 2021-05-10

**Authors:** Tamara Lang, Markus Himmelsbach, Franz Mlynek, Wolfgang Buchberger, Christian W. Klampfl

**Affiliations:** Institute of Analytical Chemistry, Johannes Kepler University, Altenberger Strasse 69, 4040 Linz, Austria

**Keywords:** Environmental analysis, Plant metabolism, Pharmaceuticals, Sartans, Drift-tube ion-mobility mass spectrometry, Plant uptake

## Abstract

In the present study, the uptake and metabolization of the sartan drug telmisartan by a series of plants was investigated. Thereby for seven potential metabolites, modifications on the telmisartan molecule such as hydroxylation and/or glycosylation could be tentatively identified. For two additional signals detected at accurate masses m/z 777.3107 and m/z 793.3096, no suggestions for molecular formulas could be made. Further investigations employing garden cress (*Lepidium sativum*) as a model plant were conducted. This was done in order to develop an analytical method allowing the detection of these substances also under environmentally relevant conditions. For this reason, the knowledge achieved from treatment of the plants with rather high concentrations of the parent drug (10 mg L^−1^) was compared with results obtained when using solutions containing telmisartan in the μg - ng L^−1 ^range. Thereby the parent drug and up to three tentative drug-related metabolites could still be detected. Finally cress was cultivated in water taken from a local waste water treatment plant effluent containing 90 ng L^−1^ of telmisartan and harvested and the cress roots were extracted. In this extract, next to the parent drug one major metabolite, namely telmisartan-glucose could be identified.

**Supplementary Information:**

The online version contains supplementary material available at 10.1007/s11356-021-14289-4.

## Introduction

Since the first successful detection of drugs (respectively drug metabolites) in the aquatic system by Hignite and Azarnoff (1977), environmental waters and in particular effluents of wastewater treatment plants (WWTP) have been frequently analyzed with respect to the presence of drug residues (for exemplary reviews, see Buchberger 2011; Cotton et al. 2016; Lindberg et al. 2014; Petrie et al. 2013; Puckowski et al. 2016; Richardson and Ternes 2014; Rzymski et al. 2017). Within the last years, a new facet related to the certainty that pharmaceuticals can be found in the effluent of WWTPs all over the world has emerged, as those waters are increasingly used for irrigation purposes in agriculture. Main reasons for this practice are rising problems with droughts paired with an increasing demand for food and feed (due to the continuous growth of the world´s population) triggering a search for new sources of water (European Commission 2013, 2008). Although such reclaimed waters used in the irrigation of plants intended for human or animal consumption must fulfill certain minimum standards (mainly with respect to microbiological and physico-chemical parameters), until now no regulation focusing on the presence of emerging contaminants (such as pharmaceuticals) exists (Alcalde-Sanz and Gawlik 2017). Furthermore, certain plants have been tested and subsequently employed for their potential in the removal of xenobiotics from wastewater in constructed wetlands (Nuel et al. 2018). All these facts have led to a growing interest in investigations dealing with the interaction between plants and these emerging contaminants added through irrigation water or contaminated fertilizer (e.g., manure from treated animals or sludge from WWTPs) (for relevant reviews, see Fu et al. 2019; Kinney and van den Heuvel 2020; Klampfl 2019; Pico et al. 2017; Reddy Pullagurala et al. 2018). Among these studies, those mainly directed towards investigating the uptake of the parent drug (or its metabolite) from water or soil, and a second type primarily devoted to research on the further biotransformation of the drug focusing on the identification of new metabolites formed within the plant can be distinguished (Klampfl 2019; Klampfl et al. 2021). Thereby, within the first group, the focus was often placed on experimental set-ups that were as much as possible resembling real environmental conditions. Therefore, from an analytical point of view, approaches suitable for ultra-trace analysis of the parent drug and sometimes its main metabolite(s) have to be pursued (for exemplary papers, see Beltrán et al. 2020; Keerthanan et al. 2021; Montemurro et al. 2017; Reddy Pullagurala et al. 2018). When the elucidation of the biological fate of the drug (after uptake) within the plant was the main goal, plants were often treated with much higher concentrations of the drug under investigation to facilitate the detection of as many metabolites as possible (for exemplary papers see, e.g., Emhofer et al. 2019; Klampfl 2019; Mlynek et al. 2020). Here the main focus was set on the tentative identification of structures and the metabolic pathways responsible for the formation of these substances. However, it should be stated that of course a series of research papers not exactly fitting into this scheme exists (e.g., Emhofer et al. 2018; Riemenschneider et al. 2017). Throughout the years, an ever-increasing plentitude of different drugs but also personal care products, summarized as pharmaceuticals and personal care products (PPCPs), was included in these studies. For about five years, the group of sartan drugs, such as valsartan or telmisartan (TEL), has been added to this portfolio with studies on the presence of these substances in environmental waters (Bayer et al. 2014; Castro et al. 2019; Giebułtowicz et al. 2016). The interaction of sartans with plants has so far only been studied in a single paper reporting the uptake and metabolization of this drug by *Salix alba* from a constructed wetland (Villette et al. 2019). Within that work, several TEL metabolites (based on hydroxylation, oxidation, and demethylation) as well as their distribution within *Salix alba* leaves using mass spectrometry imaging were described.

In the present paper, we conducted experiments using several plants grown either directly in water or hydroponically. Thereby the potential metabolites were characterized by their chromatographic behavior (retention time in reversed-phase high-performance liquid chromatography (RP-HPLC)), their accurate mass, and fragmentation in MS/MS experiments. In addition collision cross sections (^DT^CCS_N__2_) using drift-tube ion-mobility quadrupole time-of-flight mass spectrometry (DTIM-QTOF-MS) with nitrogen as drift gas were measured. Finally, garden cress (*Lepidium sativum*) was selected as a model for research directed towards setting up an analytical method allowing the investigation of TEL uptake and metabolism in plants under environmentally relevant conditions. To evaluate the proposed methodology, cress plants were cultivated in water sampled from the receiving water course of a local WWTP.

## Experimental

### Chemicals and materials

**Fig. 1:**
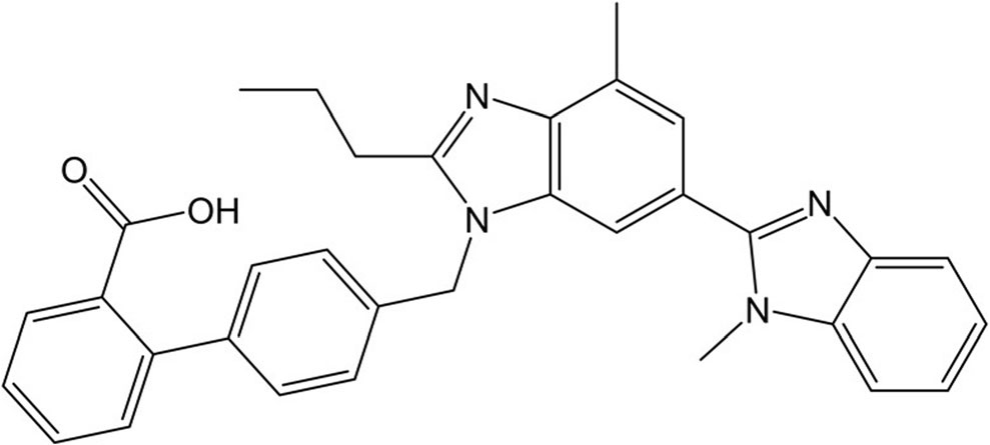
Structure of telmisartan

Telmisartan (TEL) 80 mg (for structure see Fig. 1) was obtained as pharmaceutical preparation from Ratiopharm (Ulm, Germany). One tablet containing 80 mg TEL was homogenized with mortar and pestle. Sixty milligrams of the material (corresponding to 10 mg TEL) was used to prepare a 1000 mg L^−1^ stock solution in methanol. Further solutions of 100 mg L^−1^ and 10 mg L^−1^ were prepared by diluting the stock solution with Milli-Q water. For plant treatment, the solutions were further diluted with tap water. TEL-d3 (internal standard) was supplied by Cayman Chemical Company (Michigan, USA); from this, a 10 mg L^−1^ stock solution was prepared in methanol.

Methanol and acetonitrile were delivered by VWR (Vienna, Austria). Formic acid (eluent additive for LC-MS, 98%) was purchased from Sigma-Aldrich (Steinheim, Germany). Purified water was produced by a Milli-Q water purification system (Millipore, Bedford, MA, USA).

### Plant cultivation and treatment

The cress seeds (*Lepidium sativum L.*) were supplied by Sperli (Everswinkel, Germany), cyprus grass (*Cyperus zumula*) was from OBI (Linz, Austria), pea (*Pisum sativum*, cv. Premium) from MoravoSeed CZ Corporation (Czech Republic), and maize (*Zea mays*, cv. Agnan) from Oseva Agro Brno Ltd. (Czech Republic). For detection of TEL-related metabolites, samples were cultivated under hydroponic conditions in 10 mg L^−1^ TEL solution and harvested after 7–12 days of cultivation. A more detailed description of the cultivation procedure can be found in a previously published report (Mlynek et al. 2021). Water plants, namely three-part pennywort (*Hydrocotyle cf. tripartite*) and parrot leaf (*Alternanthera reineckii*) were cultivated in 30 cm × 20 cm × 20 cm aquaria filled with tap water. For treatment, a certain amount of the plant was transferred to an Erlenmeyer flask containing in 10 mg L^−1^ TEL in tap water.

Cress was in addition cultivated in solutions of 10 μg L^−1^, 1 μg L^−1^, 100 ng L^−1^, and 10 ng L^−1^ TEL solutions and water from a WWTP effluent. For germination, seeds were soaked overnight in the corresponding growing solution and afterwards placed on cress-growing containers and humidified once a day for 2 days. During the germination period, the containers were kept in small greenhouses placed on the lab-window bench. Afterwards, the top of the greenhouse was removed in order to ensure proper air circulation. The containers were filled with the growing medium, which was replenished approximately every 2 days. This consumed in total 0.6 l for each dish.

### Preparation of plant extracts for tentative identification of metabolites

The plants were harvested after 7–12 days in total and for cress, pea and maize roots and upper part were separated. The plant material was cleaned three times with tap water and dried with paper tissue. 1.5 g (wet weight) of the plant material was transferred into a 15-mL centrifugation tube. Subsequently, 3 mL of the extraction solvent as well as three steel beads with a diameter of 5 mm was added. As extraction solvent acetonitrile/water (v/v 1/2) was used. Samples were homogenized using a ball mill (“Star Beater,” VWR, Vienna Austria) for 15 min at 20 Hz. The homogenized samples were centrifuged for 20 min at 4000 rpm. The supernatant was attained with a syringe and subsequently filtered into 1.5-mL HPLC glass vials using a 0.45-μm syringe filter. The samples were stored at −80 °C until analysis.

### Internal standard

TEL-d3 was implemented as internal standard (ISTD) for the targeted analysis. The 1000 ng L^−1^ ISTD was added to the wastewater as well as to the extracts of cress cultivated in wastewater (in the ratio ISTD/sample 1/10, v/v) directly before analysis.

### Analysis of WWTP effluent samples

0.5 L of water was filtered through a 0.45-μm filter and passed through an Oasis HLB (Waters, Milford USA) solid-phase extraction cartridge. The extract was eluted using 5 mL of MeOH which was subsequently evaporated to (almost) dryness. After re-dissolving the residue in 500 μL of purified water, the sample was analyzed using the HPLC/DTIM-QTOF-MS instrument described below.

### HPLC/DTIM-QTOF-MS and HPLC/QqQ-MS/MS instrumentation

The samples were separated by RP-HPLC using an Agilent 1260 HPLC system from Agilent Technologies (Waldbronn, Germany) equipped with a degasser, a quaternary pump, and an autosampler. The separation was performed on a Poroshell 120 EC-C18 column (3 × 150 mm, particle size 2.7 μm, Agilent) that was protected with a C18 guard column (4 × 3 mm, particle size 3 μm) from Phenomenex (Aschaffenburg, Germany).

For HPLC separation, a water/methanol gradient was applied. Starting conditions were set to 95% solvent A (water with 0.1% formic acid) and 5% solvent B (methanol). From minute 0 to 15, solvent B was increased to 100% and kept constant for 5 min. The column was re-equilibrated for 5 min, resulting in a total run time of 25 min. The flow rate was set to 0.4 mL min^−1^, the temperature of the column heater was 30 °C, and an injection volume of 20 μL was used.

For the tentative identity confirmation of metabolites, the HPLC system was hyphenated with an Agilent 6560 DTIM-QTOF LC-MS/MS (operated either in the “QTOF only” or ion-mobility MS mode) equipped with a Dual AJS ESI source (Agilent Technologies, Waldbronn, Germany).

The DTIM-QTOF-MS was tuned in the “fragile ion” mode and operated in the positive ionization mode with the following source parameters: drying gas temperature 300 °C, drying gas flow rate 10 L min^−1^, nebulizer pressure 50 psi, sheath gas temperature 300 °C, sheath gas flow rate 10 L min^−1^, capillary voltage 3500 V, nozzle voltage 1000 V, and fragmentor 425 V. For MS/MS experiments, nitrogen was used as collision gas and collision energies of 20 V and 30 V were applied.

The ^DT^CCS_N__2_ values were obtained using nitrogen as drift gas and the following parameters for the DTIM device: 4-bit multiplexing, frame rate 0.9 frames s^−1^, IM transient rate 18 transients frame^−1^, max drift time 60 ms, trap fill time 3900 μs, and trap release time 250 μs. Calibration was performed according to a “single-field” approach, allowing the determination of the ^DT^CCS_N__2_ values. In order to relate the measured drift times to known and standardized ^DT^CCS_N__2_ values of the calibrant analytes, a tune mix calibrant was measured (applying the same conditions as for the samples) before analyzing the actual sample. Drift tube parameters (for “single-field” measurements) were the following: drift tube entrance 1567 V, drift tube exit 217 V, rear funnel entrance 210.5 V, and rear funnel exit 38 V.

For the targeted analysis, an HPLC was coupled to an Agilent 6460 triple quadrupole QqQ-MS/MS (Agilent Technologies, Waldbronn, Germany) equipped with an AJS ESI source.

The QqQ MS/MS system was operated in the positive ionization mode. Applied parameters were as follows: capillary voltage 4000 V, drying gas flow rate 10 L min^−1^, drying gas temperature 300 °C, nebulizer pressure 45 psi. The optimized parameters (selected transitions, fragmentor voltages and collision energies) for the multiple reaction monitoring (MRM) mode are given in Table 1.

**Table 1:** Parameters for TEL and metabolites tentatively identified in garden cress

								MRM parameters	
Name	Formula	RT	m/z (H^+^)	CCS (Å^2^)	Mass error (ppm)	Precursor-Ion	Product-Ion	Fragmentor voltage (V)	CE (V)
TEL-OH-glc	C_39_H_40_N_4_O_8_	11.0–13.6	693.2914	231.9–233.7	−0.5770	693.3	531.2	80	50
						693.3	513.2	80	50
TEL-glc	C_39_H_40_N_4_O_7_	11.3–14.4	677.2965	254.5–259.1	−0.5906	677.3	515.2	50	20
						677.3	497.2	50	50
						677.3	276.2	50	50
TEL-glc-glc	C_45_H_50_N_4_O_12_	11.8	839.3497	273.8	0.0000	839.3	515.2	80	20
						839.3	497.2	80	50
						839.3	276.2	80	50
TEL-OH-glc-mal	C_42_H_42_N_4_O_11_	12.4	779.2924	271.1	0.2566	779.3	531.2	80	30
						779.3	513.2	80	30
Unknown I	C_43_H_44_N_4_O_11_	12.5	793.3079	271.6	0.0000	793.3	531.2	80	30
						793.3	531.2	80	30
TEL-glc-mal	C_42_H_42_N_4_O_10_	12.8–14.4	763.2985	266.6–267.3	1.5721	763.3	515.2	80	20
						763.3	497.2	80	50
						763.3	276.2	80	50
Unknown II	C_43_H_44_N_4_O_10_	13.0–13.3	777.3135	270.8–270.9	0.6432	777.3	515.2	80	20
						777.3	497.2	80	50
						777.3	276.2	80	50
TEL-OH	C_33_H_30_N_4_O_3_	13.4–13.8	531.2384	231.9–233.7	−1.1294	531.2	513.2	80	30
						531.2	292.2	80	50
						531.2	276.2	80	50
TEL	C_33_H_30_N_4_O_2_	14.0	515.2447	230.3	1.1645	515.2	497.2	251	50
						515.2	276.2	251	50
TEL-d3	C_33_H_27_D_3_N_4_O_2_	14.0	-	-		518.2	500.2	251	50
						518.2	279.2	251	50
TEL-glcA	C_39_H_38_N_4_O_8_	14.3	691.2764	256.9	0.2893	691.3	515.2	80	20
						691.3	497.2	80	50
						691.3	276.2	80	50

### Data processing

For data evaluation, Agilent MassHunter Qualitative Analysis B.07.00, MassHunter Quantitative Analysis B.10.1, MassHunter PCDL Manager B.08.00, PNNL PreProcessor (2020.03.23), and the IM-MS Browser B.10.00 were used.

A database (PCDL) was created, similar as described in a previous study (Mlynek et al. 2021). Therefore, TEL or hydroxylated TEL were in silico combined with common building blocks of phase II metabolites like glucose or malonic acid in every combination and any number possible. This database is used for screening the MS spectra of treated plant samples. The results were verified by a targeted MS/MS looking at the accurate masses and fragmentation pattern. With the fragmentation pattern, possible sum formulas for metabolites were reconstructed from mass losses.

Ion-mobility (IM) data files were first demultiplexed using the PNNL PreProcessor software. Afterwards, the data were calibrated with the recorded single field tune using IM-MS browser. The drift times and the ^DT^CCS_N__2_ were determined by first performing a feature extraction (Find features IMFE). Parameters were processing chromatographic, isotope model common organic molecules, limit charge state z ≤ 1, ion intensity ≥ 1. The drift times and the collision cross sections of all features were automatically determined by the software. Within all the features found by the software, the analytes of interest were identified according to their m/z values and their retention times.

## Results and discussion

### Tentative identification of TEL metabolites by DTIM-QTOF-MS

Extracts from plants cultivated in the presence of 10 mg L^−1^ TEL, either hydroponically (pea, cress, maize) or directly in water (pennywort, parrot leaf), were screened for the presence of potential TEL-related metabolites using HPLC-DTIM-QTOF-MS. Employing the lab-made database, altogether nine TEL-derived substances were found in addition to the parent drug, whereby positive hits were further confirmed by MS/MS measurements. From published work, it was known that upon uptake by plants TEL can be hydroxylated (either one or two times), demethylated, or decarboxylated (Villette et al. 2019). Based on these findings, we could propose suggestions for tentative sum formulas for seven hits — whereas in two cases (i.e., m/z 777.3107 and m/z 793.3096), no proposal for a tentative structure could be made. The tentatively identified compounds comprise phase I + II metabolites resulting from the addition of glucose or malonic acid (or a combination of both) either to TEL (most probably to the carboxylate group) or to hydroxylated TEL (TEL-OH), where the hydroxyl group is the most probable site for conjugation. An overview of metabolites detected in these plants is given in Table 2.

**Table 2:** Presence of TEL and tentatively identified metabolites in investigated plants

Name	Formula	m/z (H^+^)	Garden cress	Maize	Pea	Three-part pennywort	Parrot leave
TEL-OH-glc	C_39_H_40_N_4_O_8_	693.2914	Yes	Yes	Yes	Yes	X
TEL-glc	C_39_H_40_N_4_O_7_	677.2965	Yes	Yes	Yes	Yes	Yes
TEL-glc-glc	C_45_H_50_N_4_O_12_	839.3497	Yes	Yes	X	X	X
TEL-OH-glc-mal	C_42_H_42_N_4_O_11_	779.2924	Yes	Yes	X	Yes	X
Unknown I	C_43_H_44_N_4_O_11_	793.3079	Yes	Yes	Yes	Yes	Yes
TEL-glc-mal	C_42_H_42_N_4_O_10_	763.2985	Yes	Yes	Yes	Yes	X
Unknown II	C_43_H_44_N_4_O_10_	777.3135	Yes	X	X	X	X
TEL-OH	C_33_H_30_N_4_O_3_	531.2384	Yes	Yes	Yes	Yes	Yes
TEL	C_33_H_30_N_4_O_2_	515.2447	Yes	Yes	Yes	Yes	Yes
TEL-glcA	C_39_H_38_N_4_O_8_	691.2764	Yes	X	X	X	X

*X*, not detected

For further studies, garden cress was selected as the best suited model plant. As the basic concept of the present study was to allow the analysis of plants grown under environmentally relevant conditions besides a more in-depth investigation on the presence of TEL-related substances in the extract, also a MRM method was developed on a QqQ-MS/MS instrument. Relevant information on the outcome of these investigations is summarized in Table 1. Regarding relative intensities (due to the lack of standard substances, only signal areas could be compared) of metabolite signals, TEL-glucose (TEL-glc) could be identified as the major metabolite, showing an abundance between 80 and 100% compared to that of TEL. The peak area for TEL combined with glucose and malonic acid (mal) was between 14 and 20% of that obtained for the parent drug. For the other compounds, substantially smaller peaks with areas of 1% and below (compared to TEL) were registered.

**Fig. 2:**
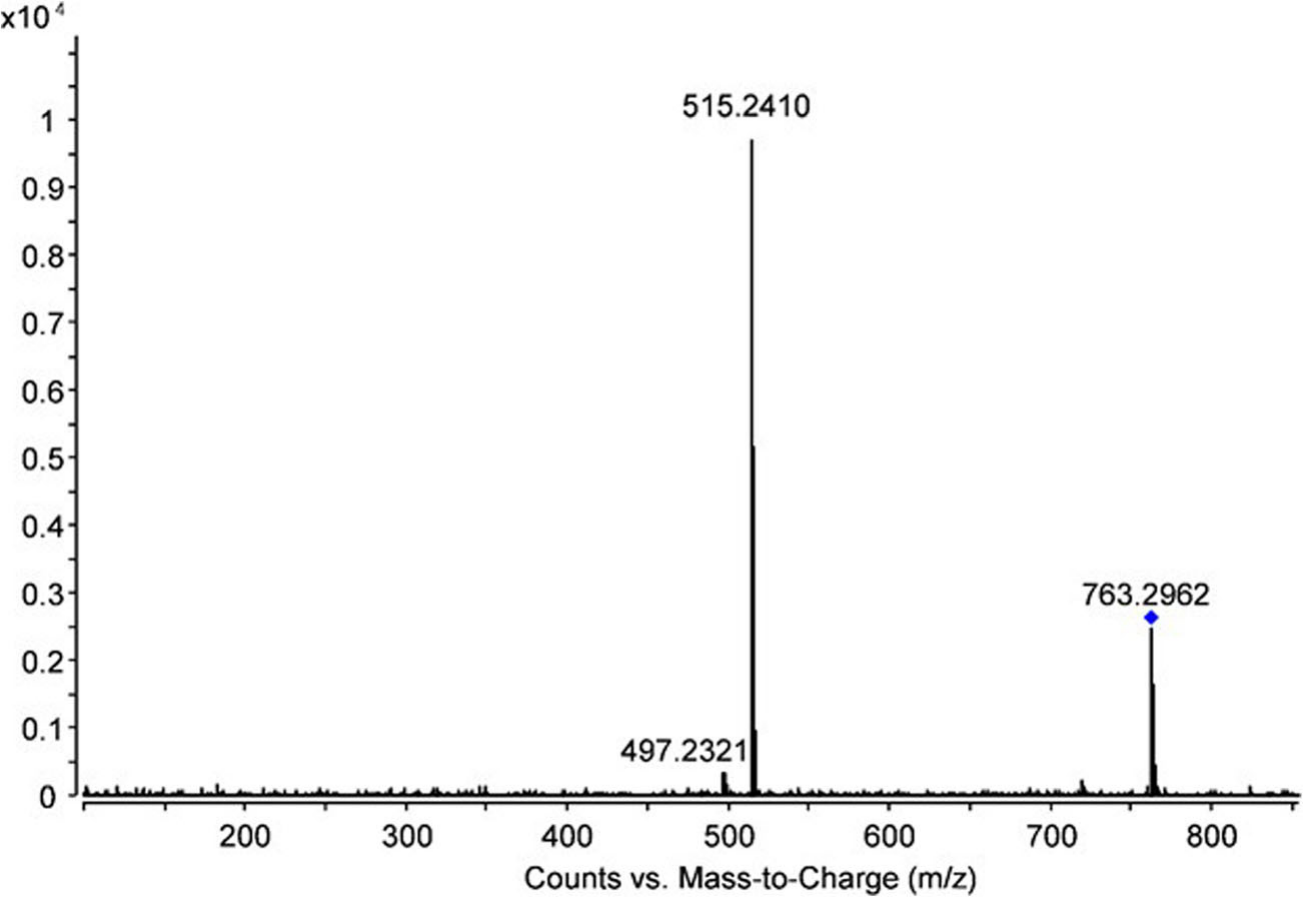
MS/MS spectrum for TEL-glc-mal. Isolation width 4 u, collision energy 20 V

**Fig. 3:**
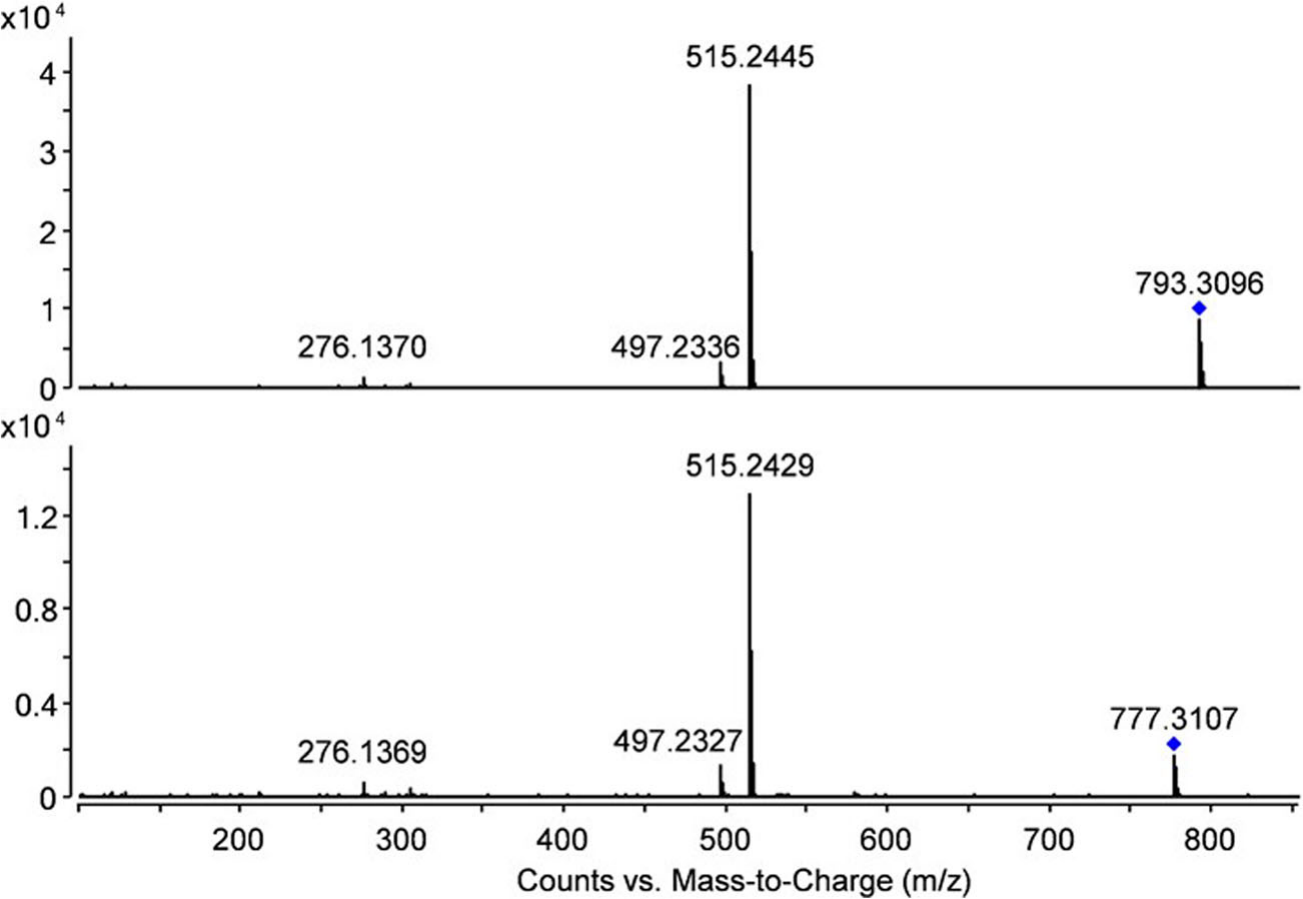
MS/MS spectrum for unknown metabolite I (upper trace) and unknown metabolite II (lower trace). Isolation width 4 u, collision energy 20 V

For an overview of retention time ranges, accurate mass (of the protonated species), mass error, and ^DT^CCS_N__2_, see Table 1. In this table, no exact retention times but retention time ranges are provided as for several compounds more than one signal with identical accurate mass and MS/MS spectrum but slightly different retention time and also slightly different ^DT^CCS_N__2_ was detected. This was in accordance with previous observations and can be explained by the presence of isomers showing variations in the three-dimensional structure of the molecules, thereby influencing both the chromatographic behavior and the drift times in the ion-mobility device (Mlynek et al. 2020). An exemplary MS/MS spectrum for TEL-glc-mal, a metabolite detected in the cress extracts can be seen in Fig. 2. For the two unidentified metabolites, accurate masses of m/z 777.3107 and m/z 793.3096 were determined. Using the PCDL, the following suggestions were obtained: TEL with glucose and malonic acid with an additional methyl group or succinic instead of malonic acid for the first mass, and the same composition but originating from TEL-OH instead of TEL for the second. The ∆ m/z of 15.9989 suggesting the presence of an additional oxygen in the second molecule can be seen as a further hint strengthening this proposal. Unfortunately the latter assumption is not bolstered by the MS/MS data as in both cases, employing collision energies of 20 V, a direct transition to TEL (and not to TEL-OH) with subsequent loss of a water molecule was observed (see Fig. 3). These data strongly suggested that both tentative metabolites originated from TEL and the additional oxygen was part of the moiety attached to a TEL molecule. A comparison of the MS/MS spectra obtained for the two unknown metabolites is depicted in Fig. 3.

### Targeted analysis of the parent drugs and their metabolites by implementing an HPLC-QqQ-MS/MS method

Based on the results described in the “Tentative identification of TEL metabolites by DTIM-QTOF-MS” section, investigations on the trace level detection of TEL and its tentative metabolites in extracts from cress plants were performed. The main goal of these experiments was to find out whether the developed approach might be used for plantlets grown in actual reclaimed waters or waters drawn from the effluents of WWTPs. Therefore, several batches of cress plants were cultivated in parallel hydroponically using water containing TEL at levels between 10 μg L^−1^ (3 orders of magnitude less than the concentration level employed for the search for potential metabolites) and 10 ng L^−1^. Thereby the parent drug and TEL-glc were found in all plants whereas TEL-OH-glc was found in the plants cultivated in the10 μg L^−1^ and 1 μg L^−1^ solution only. TEL-OH could exclusively be detected in the cress from the 10 μg L^−1^ growing medium. To evaluate parameters relevant for quantitative analysis of TEL, two calibration curves (one in water and one in cress extract matrix) were constructed between 20 and 200 ng L^−1^. Both showed a correlation coefficient of 0.99 or better. The fact that a slope of around 16.200 was achieved for the standards in water compared to around 11.700 for the matrix matched calibration suggested the presence of ionization suppression in the ESI source caused by plant matrix compounds. LODs for TEL in water and cress matrix were both found to be 9 ng L^−1^. In order to further evaluate the analytical procedure, five sets of plants were cultivated, harvested, and spiked with TEL and plant extracts were prepared on three consecutive days. These extracts were subsequently analyzed three times each by HPLC-QqQ-MS/MS. These experiments led to an average recovery of 69% with a relative standard deviation of (RSD) 8.7% on day one, 68% with a RSD of 8.6% on day two, and 70% with a RSD of 8.8% on day three for TEL.

### Analysis of garden cress cultivated in water from a WWTP effluent

A water sample was drawn from the effluent of a WWTP and analyzed qualitatively (and also quantitatively for TEL) using HPLC-QTOF-MS after appropriate sample pre-treatment (for exact procedure see the “HPLC/DTIM-QTOF-MS and HPLC/QqQ-MS/MS instrumentation” section). An overview of the pharmaceuticals detected in this water sample (employing a semi-targeted approach) is provided in Table S1. HPLC-QqQ-MS analysis revealed a TEL content of 90 ng L^−1^ in the effluent sample that was further employed for growing the cress plantlets hydroponically. The water sample was also checked for the presence of TEL-related metabolites (in particular TEL-OH) but none of them could be detected. Subsequently, cress plantlets were grown in the water from the WWTP effluent and harvested, and in a first step extracts from the plants were screened employing the RP-HPLC MRM QqQ MS method already used for the experiments described in the “Targeted analysis of the parent drugs and their metabolites by implementing an HPLC-QqQ-MS/MS method” section. Thereby, as was expected, only TEL and TEL-glc could be detected. All other metabolites were below or very close to the LOD, leading to m/z traces that could not be quantified properly. The TEL concentration determined in three independently grown batches of cress, employing a matrix matched calibration curve, was 390 pg g^−1^ for cress roots (wet weight) with a relative standard deviation (RSD) of 25%. In this context, it must be taken into account that apart from the analytical methodology employed, also biological variations contributed to this RSD. For comparison 90 μL of the cress extract was spiked with 10 μL of a 1000 ng L^−1^ TEL-d3 standard solution to perform quantitative analysis also using the standard addition method. Evaluation of these experiments led to a TEL concentration of 550 pg per g cress with an RSD of 24%. A different situation was encountered for TEL-glc as no standard for quantitation was available. Here only peak areas could be compared. The fact that for the plants grown in a 100 ng L^−1^ solution of TEL (a concentration very close to the one in the WWTP effluent), a 50% higher peak area was registered for TEL-glc than for those grown in water from a WWTP effluent might be explained by the presence of other xenobiotics in the latter, partially utilizing the same biological pathways required for glycosylation of the parent substance.

## Conclusions

In the present work, we managed to tentatively identify eight TEL-related metabolites formed in three different plants cultivated hydroponically and in two water plants. In both cases, growing media containing TEL in the mg L^−1^ range were used. Based on these findings, further investigations were conducted, using garden cress as a model plant, focusing on the possibility to transfer this knowledge also to environmentally relevant samples commonly in contact with emerging contaminants like TEL in much lower concentrations. The approach followed was to cultivate garden cress hydroponically in a water sample drawn from a WWTP effluent. In the corresponding samples, TEL as well as one major metabolite (TEL-glc) could be identified and TEL quantified using a matrix matched calibration curve as well as the standard addition method employing a deuterated TEL standard. As WWTP effluent samples commonly contain a plethora of emerging pollutants in concentrations up to the high ng L^−1^ range, further work will be directed towards synergetic effects with respect to uptake and metabolization of widely used drugs in such plant models.

## Supplementary Information

ESM 1 (DOCX 12 kb)

## Data Availability

All data and materials are included in this published article.
