# Supplementary material for: Uptake and bio-transformation of telmisartan by cress (Lepidium sativum) from sewage treatment plant effluents using high-performance liquid chromatography/drift-tube ion-mobility quadrupole time-of-flight mass spectrometry
Source: Environ Sci Pollut Res Int. 2021 May 10;28(36):50790–8. doi: 10.1007/s11356-021-14289-4 (PMC8445863; doi:10.1007/s11356-021-14289-4)
Supplement: Supplementary file 1 — (DOCX 12 kb) [file 11356_2021_14289_MOESM1_ESM.docx]

Table S1: Drug residues found in the water sample used for growing the cress plantlets hydroponically

| Acetylsalicylic acid | Clindamycin | Naproxen |
| --- | --- | --- |
| Amoxicillin | Diclofenac | Oxazepam |
| Atenolol | Ibuprofen | Sulfamethazine |
| Bezafibrate | Irbesartan | Telmisartan |
| Bisoprolol | Lamotrigine | Tramadol |
| Candesartan | Lidocaine | Trimethoprim |
| Carbamazepine | Losartan | Valsartan |
| Cetirizine | Mefenamic acid | Venlafaxine |
| Citalopram | Metoprolol |  |
